# Prescription Department

**Published:** 1892-02

**Authors:** 


ENLARGED SPLEEN.—

℞. Quinæ sulph.,

Ferri redacti, aa dr. j.

Podophyllin, - gr. v.

Syr. symphl., - q. s.

M. ft. pil. No. XXX.

Sig. One after each regular meal. If they should act a little too freely on the bowels, omit taking the one after dinner. Be governed by the way they act on the bowels.—*The Clinique.*

NEURALGIA.—

℞. Aconitæ, gr. iv.
Veratriæ, gr. xv.
Glycerinæ dr. ij.
Cerati, dr. vj.

M. Sig. To be rubbed over the parts; care should be taken to see that there is no abrasion of the skin. Among the many local applications, I have found this one best adapted for general use.

DRY BRONCHIAL CATARRH.—

℞. Potassii iodidi, gr. v. x.
Eucalyptol (Sanders), gtt. j. v.

M. Sig. One dose. Repeat thrice daily.—*Memphis Med. Monthly.*

AMENORRHŒA.—

The following is recommended as a reliable emmenagogue in many cases of functional amenorrhœa:

℞. Bichloride of mercury,
Arsenite of sodium, aa. gr. iij.
Sulphate of strychnine, gr. iss.
Carbonate of potassium,
Sulphate of iron, aa gr. xlv.

Mix and divide into sixty pills.

Sig. One pill after each meal.—*Revue Med. Chir. Mal Femmes.*

INFANTILE DIARRHŒA.—

℞. Aq. calcis, dr. vi.
Syr. rhei. ar., oz. j.
Tr. opii camph, oz. ss.
Tr. cardam co., oz. ss.

M. Sig. One teaspoonful, often, to children under one or two years, afflicted with sour stomach, attended with diarrhœa, vomiting, etc.—Ex.

OPIUM HABIT.—

A favorite prescription for the opium habit has been:

℞. Hydrochlorate of cocaine, grs. v.
Neurosine, oz. viij.

M. Sig. Teaspoonful four times a day.

IODOFORM IN ACUTE GONORRHŒA.—
Iodoform, 2 1-2 drs.
Sweet-almond oil, 2 fl. oz.
Vanillin, or cumarin, enough to flavor.

For urethral injection.

2 fl. drs. are injected 3 times a day —the patient in dorsal decubitas—and retain 10-20 minutes. The first injection should be made by the physician. According to Thiery, pain disappears after the first injection, and cure results in 12 days.—*Ex.*

IRRITABLE COUGH.—
℞. Acetanalid, dr. ij.
Gum Acacia, dr. ij.
Syr. Ipecac, oz. ss.
Neurosine, oz. vj.

M. Sig. Teaspoonful in a little water every two or three hours.

ASTHMA.—
℞. Antipyrin, dr. ij.
Neurosine, oz. iv.
Syr yerba santa aromatic, q. s., oz. vj.

M. Sig. Dessertspoonful every two or three hours in a little water until relieved.

NASO-PHARYNGEAL CATARRH.—
First cleanse parts with peroxide of hydrogen, diluted sufficiently, and then apply the following with spray:
℞. Sodii boro-benzoat.
Fld. ext. hydrastis, aa oz. j.
Glycerini, oz. j.
Acidi carbolici, m xx.
Aquæ camphoræ, oz. vj.
Aquæ, oz. vj.

M. Sig. Use three times per day. —*Willis, Canada Lancet.*

ACUTE TONSILITIS.—

Dr. M. A. Martin (*Lo Sperimentale*, No. 21, 1891) speaks highly of the following:

℞. Acid carbolic, Camphoræ } aa gm. i (mxv)

Glycerin, Aq. destillat. } aa gms. 50 (fl. oz. jss).

Three or four applications a day.—*Ex.*

GASTRIC IRRITABILITY.—

℞. Bis. trisnitrat., gr. x.

Pot. bicarb., gr. v.

Mist. acacia, dr. j.

Infus. columbæ, oz. j.

M. Sig. Three times a day before food.—*Ex.*

